# Lead field theory provides a powerful tool for designing microelectrode array impedance measurements for biological cell detection and observation

**DOI:** 10.1186/s12938-017-0372-5

**Published:** 2017-06-26

**Authors:** Marcel Böttrich, Jarno M. A. Tanskanen, Jari A. K. Hyttinen

**Affiliations:** 1 Biosignal Processing Group, Department of Computer Science and Automation, Institute of Biomedical Engineering and Informatics, Technische Universität Ilmenau, Gustav-Kirchhoff-Straße 2, 98693 Ilmenau, Germany; 2 Computational Biophysics and Imaging Group, BioMediTech Institute and Faculty of Biomedical Sciences and Engineering, Tampere University of Technology, Lääkärinkatu 1, 33520 Tampere, Finland

**Keywords:** Bioimpedance, Biological cells, Finite element analysis, Impedance spectroscopy, Microelectrodes, Sensor arrays

## Abstract

**Background:**

Our aim is to introduce a method to enhance the design process of microelectrode array (MEA) based electric bioimpedance measurement systems for improved detection and viability assessment of living cells and tissues. We propose the application of electromagnetic lead field theory and reciprocity for MEA design and measurement result interpretation. Further, we simulated impedance spectroscopy (IS) with two- and four-electrode setups and a biological cell to illustrate the tool in the assessment of the capabilities of given MEA electrode constellations for detecting cells on or in the vicinity of the microelectrodes.

**Results:**

The results show the power of the lead field theory in electromagnetic simulations of cell–microelectrode systems depicting the fundamental differences of two- and four-electrode IS measurement configurations to detect cells. Accordingly, the use in MEA system design is demonstrated by assessing the differences between the two- and four-electrode IS configurations. Further, our results show how cells affect the lead fields in these MEA system, and how we can utilize the differences of the two- and four-electrode setups in cell detection. The COMSOL simulator model is provided freely in public domain as open source.

**Conclusions:**

Lead field theory can be successfully applied in MEA design for the IS based assessment of biological cells providing the necessary visualization and insight for MEA design. The proposed method is expected to enhance the design and usability of automated cell and tissue manipulation systems required for bioreactors, which are intended for the automated production of cell and tissue grafts for medical purposes. MEA systems are also intended for toxicology to assess the effects of chemicals on living cells. Our results demonstrate that lead field concept is expected to enhance also the development of such methods and devices.

## Background

Microelectrode arrays (MEAs) are widely used to observe the electrophysiological activity of cells, from single-cell activity to network function [1, 2]. Electrical contact between a cell and a microelectrode affects the properties of the measured electrical signals significantly, including the shape of the waveforms [3, 4]. It has been shown that the impedance spectrum is a measure of cell–electrode junction quality [5]. Also, the knowledge of the location of adherent or nearby cells would be helpful in identifying the bioelectric sources, e.g., in neuronal action potential analysis, and in designing bioelectric systems for the detection and assessment of cells and tissues. These developments are crucial for the development of automated cell and tissue handling and assessment in future bioreactors for automated cell and tissue craft production for medical uses. Also, the automated assessment of the effects of chemicals on the cells and tissues is important for the development of automated drug and toxicity screening systems.

IS can be used to observe changes in the viability of biological cells in the vicinity of the electrodes, and in the adherence of the cells to electrodes [6–8]. Giaever and Keese [6, 9] used relatively large electrodes compared to the cell size when they first employed IS to observe dynamic cellular processes such as cell proliferation and motility. Amongst others, Buitenweg et al. [3], Huang et al. [10], and Yufera et al. [8] extended the concept, and studied the influence of changes in the shapes, compositions, and locations of the cells to the impedance spectrum using cell-sized microelectrodes. In general, two-electrode impedance measurement setups have been employed in analyzing adhered cells.

Huang et al. [10] performed simulations of the cell–electrode gap. They demonstrated that decreasing the distance between a cell surface and an electrode, leads to an increase in impedance. Buitenweg et al. [3] measured the impedances of microelectrodes with and without adhered cells, and noticed significant changes in the impedance loci. Yufera et al. [11] focused on cell–electrode overlap, and developed models for biometry applications, where impedance was used in determining cell size and count, and to observe cell growth with respect to different doses of drugs. Their results indicated that cells adherent to electrodes can be detected, however, the distance for detection was very small. Moreover, IS has been used to study the effects of pharmaceuticals and toxins to the passive electric properties of cells [11], and IS-based impedance tomography has been proposed for obtaining a spatial map of cell clusters in a culture [12]. Flow cytometry of cells in microfluidic devices is another field of application [13]; here, the challenge is the detection of cells flowing past the electrodes. Impedance changes also carry information about cell state, and can thus be used to classify them [14], in addition to merely counting the cells.

In IS in general, the model of the measurement environment at hand, including the electrode setup and complex interface, is needed to interpret the recorded impedance spectrum. 3D finite element method (FEM) simulation can directly connect such a model with the measurement environment, allowing us to analyze the recorded data with respect to the measurement geometry. Realistic models with proper impedance characteristics of the electrodes, cell culture medium, and cells, need to be employed to draw proper conclusions [11]. To this end, FEM and equivalent circuit models have been used to analyze cell–electrode connections affecting the electrical stimulation of cells [3], cell growth [10], and cell–electrode interfaces in general [15, 16]. All the past simulations have been based on direct simulation of impedance changes in medium with complex bioelectric cell models. Such simulations provide predictions of measurement values, but do not provide much insight in the properties of the measurement systems themselves. To study various measurement arrangements in relation with biological matter, new simulation methods, such as proposed in this paper, are called for.

The lead field concept was first formulated for bioelectric measurements by McFee et al. [17]. In this paper, we propose the utilization of the lead field concept of impedance sensitivity distribution analysis to study various impedance and IS measurement arrangements for the assessment of biological matter. Visualization of the sensitivity of an IS measurement configuration on MEAs was originally introduced by Geselowitz [18]. The lead field concept was used first in a FEM model by Hyttinen et al. [19], in the simulations of impedance measurements by Kauppinen et al. [20], and in impedance tomography by Kauppinen et al. [21]. For more on bioelectromagnetism and its history, see [22].

To demonstrate the applicability of the lead field concept in MEA system design, we used the lead field and reciprocal theorem to simulate the lead fields, i.e., the sensitivity distributions of MEA electric bioimpedance measurement setups. The method is demonstrated with simulations of a four-electrode MEA IS setup with and without a cell model, which are compared with the corresponding simulations of the established two-electrode measurement configuration. Further, we show how these methods can be used to visualize how the adhered and non-adherent cells change the setup behavior, and how the two- and four-electrode systems can be utilized in cell detection.

## Methods

The FEM models of the complex electric impedance of microelectrodes, cells, and the culture medium were developed and implemented in COMSOL Multiphysics (COMSOL, Inc., Burlington, MA, USA) to compute the sensitivity fields based on the lead field theory for two- and four-electrode configurations with both adherent and non-adherent cells, and to analyze MEA IS recordings. With the model, we demonstrate the usefulness of the computed sensitivity distributions in the analysis of the effects of local electrical property changes, such as the varying location of a cell with respect to the electrodes. The sensitivity fields are used to visualize measurement setup properties, and to assess the measurement results. This approach enables us to determine the effects of small intra- and extracellular conductivity changes to the impedance spectrum without or before actually performing measurements. The impedances are also computed directly, as if they were measured. The results of the lead field analysis and direct impedance computations are compared to demonstrate the practical application of the lead field approach for measuring adhered cells or cells in cytometry applications, and to demonstrate the capabilities and differences of the two- and four-electrode MEA IS systems.

Our simulation model is freely available in the COMSOL Model Exchange (see “Availability of data and materials” section). The provided simulation model consists of the models of the four-electrode system and a cell, and it can be easily converted into the corresponding two-electrode system model.

### Lead field and reciprocity theory in MEA impedance measurements

The measured impedance depends on the conductivity of the volume conductor surrounding the electrodes, and on all the objects located in the current field. Geselowitz [18] proposed that a change in the impedance *Z* caused by objects in volume *V* with conductance σ was given by

1 $$\Delta Z = \int\limits_{V} {\frac{S}{\Delta \sigma }dV} ,$$

where *S* is the sensitivity of the measurement setup, and Δ denotes a change in the respective quantity. Geselowitz [18] showed that the lead field of a measurement setup can be obtained by computing the current field of the current feeding electrodes and the reciprocal field of the measurement electrodes. In the four-electrode measurement system (c.f. Fig. 3a), the lead fields are obtained by applying a unit current to the current feeding electrode pair, and to a separate field potential measurement electrode pair, i.e., the current injection and voltage measurement current density fields are different. In the two-electrode configuration (c.f. Fig. 3b), the one electrode pair is used for both current feeding and potential measurement, and thus these two lead fields are identical. The lead field of an impedance measurement can be obtained by calculating the dot product of these two fields, resulting in a scalar sensitivity field *S*, which describes the scalar sensitivity field of the impedance measurement in the volume conductor:

2 $$S = {\mathbf{J}}_{{{\mathbf{LE}}}} \cdot{\mathbf{J}}_{{{\mathbf{LI}}}} ,$$

where **J**_**LI**_ is the current field in the volume conductor caused by the unit current applied to the current electrodes, and **J**_**LE**_ is the current field in the volume conductor generated by a unit current applied to the voltage measurement leads [23]. *S* (2) can by positive, negative, or zero, depending on the angle between the two current fields. If the location, conductivity, or geometry of an object, or the locations of the electrodes change in the measurement setup, the lead field and the measured impedance change accordingly. For electrode constellation design, it is worth noting that in the areas of *S* = 0, a slight change of conductance does not notably affect the impedance. A decrease in impedance in the areas of positive *S* decreases the measured impedance, and a similar change in the areas of negative *S* increases impedance [23], given that the impedivity of the medium is constant.

The sensitivity results were computed for partial volumes representing a cell at a number of positions. Normalized integral sensitivity *S*_*normal*_ was obtained by point-wise integration of the sensitivity values of the mesh elements included in the partial volume, and by thereafter normalizing with respect to the volume and computed global peak sensitivity.

It is to be noted that for the four-electrode system, the map of transfer impedance volume density is a product of the local sensitivity and the local impedivity. Here, local sensitivity depends on the electrode constellation, and the impedivities have been assumed constant in the medium, and within a cell. Thus, there, a change in sensitivity results in a directly proportional change in the impedance.

### FEM modeling of MEA electrodes

**Fig. 1:**
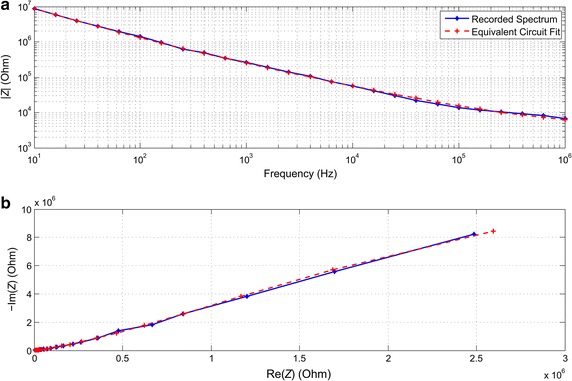
Measured microelectrode impedance spectrum (**a**) and locus (**b**), and the corresponding equivalent circuit fit. The measurements were made from commercial microelectrodes with titanium nitride surface and a diameter of 30 µm. The equivalent circuit fit was created by using the equivalent circuit shown in Fig. 2

Our FEM model included models of complex impedances of cells and microelectrodes. To obtain the electric properties of microelectrodes, microelectrode impedance spectra were measured with Solartron 1260A Impedance/Gain-phase Analyzer (Solartron Analytical, Hampshire, UK) connected to Solartron 1294A Impedance Interface, whose non-human interface was utilized. The Solartron impedance interface was connected to a standard MEA with titanium nitride coated titanium microelectrodes of 30 µm in diameter (MEA model: 60MEA200/30iR-Ti, Multi Channel Systems MCS GmbH (MCS), Reutlingen, Germany) using a contacting adapter (model: MEA1060-INV-CA, MCS). A two-electrode measurement configuration was used to obtain the characteristics of the microelectrodes. One microelectrode was used to feed a sine current with an amplitude of 1 mA, and a neighboring microelectrode was connected to ground. Impedance was determined by measuring the resulting voltage over the same two electrodes. To obtain the impedance spectrum, impedance was measured at 26 frequencies distributed logarithmically between 10 Hz and 1 MHz. The measurements were performed in phosphate buffered saline solution (PBS) with conductance 1.57 S/m and relative permittivity 77 [24]. Since the properties of culture media vary according to cells and tissues concerned, PBS was selected to serve as a generally available reference. The choice of medium may affect the absolute magnitudes of results but not the qualitative results and conclusions. An experimentally recorded impedance spectrum of a commercial MCS microelectrode is shown in Fig. 1.

**Fig. 2:**
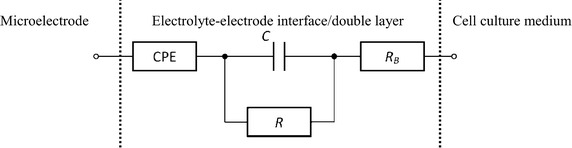
Equivalent circuit of a microelectrode in medium, motivated by [15, 26]. The model consists of a CPE and an RC element (*R* and *C*) to model the electrode–electrolyte interface. The serial resistor *R*_*B*_ models the resistance of the bulk culture medium

The equivalent circuit of an electrode in medium is shown in Fig. 2. We assume that all the microelectrodes have the equal characteristics. As proposed by McAdams et al. [25] and Buitenweg et al. [3], a constant phase element (CPE) was used to model the electrode–electrolyte interface. Here, cell culture medium acts as the electrolyte. In the CPE transfer function (3), *CPE*_*T*_ and *CPE*_*P*_ are a scaling factor and an exponent, respectively. To obtain a better fit, capacitance *C*, parallel with resistance *R*, was added in series (3) (Fig. 2). Series resistance *R*_*B*_ was used to model the bulk medium. For the model in Fig. 2, the impedance of a microelectrode is thus given by

3 $$Z_{el} = Z_{RC} + Z_{CPE} = \frac{R}{1 + j\omega RC} + \frac{1}{{CPE_{T} (j\omega )^{{CPE_{P} }} }} ,$$

where *R*, *Z*_*C*_ and *Z*_*CPE*_ are the charge transfer resistance, and the impedances of the capacitance *C* and CPE, respectively. The transfer function of the circuit (3) was implemented as contact impedance in COMSOL Multiphysics.

The equivalent circuit fit was performed with ZView^®^ (Scribner Associates Inc., Southern Pines, USA). For *CPE*_*T*_ and *CPE*_*P*_, the values of 2.819 × 10^8^ and 0.595 were obtained, respectively. The electric double layer at the interface between the electrode surface and cell culture medium (Fig. 2) was represented by *R* = 46.26 MΩ and *C* = 2.59 nF. *R*_*B*_ was set to 3.789 kΩ. The FEM model of the medium was represented by the PBS equivalent given above.

**Fig. 3:**
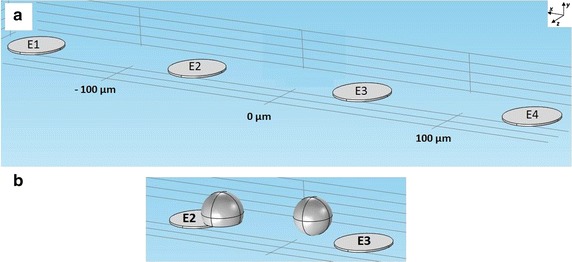
Four- (**a**) and two- (**b**) electrode systems illustrated with two possible cell models (**b**). **a** The model of the impedance measurement system with four electrodes (E1, E2, E3, and E4). **b** An adherent cell partially on top of E2 and a non-adherent cell between E2 and E3. In two-electrode system simulations (**b**), E2 and E3 were the current feeding and counter electrode, respectively. In four-electrode system simulations (**a**), E1 and E4 were the current feeding and counter electrode, respectively, and E2 and E3 were the recording electrodes. The coordinate system used in the paper is shown in **a**

We constructed a 3D model with planar electrodes on the surface of a substrate, as in the actual MEAs, with a cell in various locations in the vicinity of the electrodes (Fig. 3). In the two-electrode impedance measurement simulations, two microelectrodes (E2 and E3 in Fig. 3b) were arranged symmetrically around the origin along the horizontal axis at 50 and −50 µm. In four-electrode simulations, additional two excitation electrodes (E1 and E4 in Fig. 3a) were placed in line at 150 and −150 µm. For the simulations, ideal instrumentation with zero impedance of the current output, and infinite impedance of the recording amplifier, was assumed.

The microelectrode model implemented in COMSOL Multiphysics was scaled up by factor of 10 to account for surface roughness of the titanium nitride coated microelectrodes (e.g., MCS, model 60MEA200/30iR-Ti). To the best of our knowledge, the ratio of effective surface areas between a flat microelectrode and a rough sputtered titanium nitride coated microelectrode is not readily available in the literature. However, since COMSOL assumes perfectly flat surfaces, it was necessary to apply a scaling factor account for the larger effective surface area. This scaling resulted in a better match between the simulated and measured impedance spectra. Scaling decreased the offset impedance, and maintained the basic characteristics of the spectrum, i.e., the qualitative results do not depend on the scaling.

### FEM modeling of cells

A non-adherent cell was modeled as a sphere, and an adherent cell by a spherical cap, with the center of the sphere at different positions denoted by (*x*_0_, *y*_0_, *z*_0_) (c.f., Fig. 3a). The cell model was motivated by the work of Buitenweg et al. [4]. The typical radius of vertebral neurons, such as cortical neurons, dorsal root ganglion, and spinal cord neurons is between 7 and 20 μm [4, 27]. Here, we assumed a non-adherent cell radius of *r*_*0*_ = 10 μm. Cell volume was assumed not to change when the cell adhered to the MEA surface. Thus, the volume of the non-adherent cell *V*_*Sphere*_ was set equal to the volume of the spherical cap *V*_*Calotte*_ modeling the adherent cell (4), and the radius of the spherical cap was calculated by (4) and (5), where *h* is the height and *r* the radius of the spherical cap. (4) and (5) are valid for an orthogonal distance *y*_0_ > 0 from the MEA base plate, i.e., above the MEA substrate or an electrode. The gap, which occurs in real measurement setups between the electrode or substrate and a cell, was set to 500 nm, and neglected in radius calculations.

4 $$V_{Sphere} = \frac{4}{3}\pi r_{0}^{3} = V_{Calotte} = \frac{{h^{2} \pi }}{3}(3r - h)$$

5 $$r = \frac{4}{{3h^{2} }}r_{0}^{3} + \frac{h}{3} ,\;\;\;{\text{where}} \;h = r_{0} + y_{0}$$

Simulations were performed with orthogonal cell-substrate or cell–electrode distances *y*_0_ of 5, 10 and 15 µm, with the cell in different horizontal positions *x*_0_ between 0 and 200 µm (for the coordinate system directions, see Fig. 3a). Contact impedance was defined on the surface to model the cell membrane as a thin layer. Regarding the passive electric properties of the cell membrane, the parallel RC element modeled the cell surface conductance of 1 mS/cm^2^ and capacitance of 1 µF/cm^2^. The conductivity and relative permittivity of the medium were set to 3 S/m and 80, respectively [3, 10, 28].

The geometry of also the cell was scaled up by factor 10 to maintain the geometric relations between the cell and electrodes. The overall impedance of the cell was also scaled not to change the electric characteristic of the cell with respect to the measurement system. In a system like presented in this paper, the scaling does not affect the results, but leads to a more realistic model of the MEA electrodes.

### FEM simulation setup

The electric currents physics of COMSOL Multiphysics was used to calculate the electric fields and current density distributions in the volume conductors associated with the analyzed electrode configurations. A current *I*_*T*_ was fed in the volume via terminals at the bottom of the corresponding microelectrodes, while the counter microelectrode was set to ground. All other boundaries of the bulk medium were set to insulation. In the two-electrode configuration, E2 was the excitation, and E3 the counter electrode (c.f., Fig. 3b). The physics interface allows direct determination of the ratio of the voltage and current recorded across the electrodes. The four-electrode system was modeled using E1 as the excitation and E4 as the counter electrode (c.f., Fig. 3a). Voltages *U*_*T*2_ and *U*_*T*3_ were recorded at E2 and E3, whose bottom surfaces were set to insulation with zero terminal current. With this setup, the impedance was calculated by

6 $$Z = \frac{{U_{T2} - U_{T3} }}{{I_{T} }}.$$

All simulations were performed in frequency domain to generate impedance spectra. Impedances were simulated at 18 frequencies: 10, 20, 30, 40, 50, 80, 100, 200, 500 Hz, 1, 2, 5, 10, 20, 50, 100, 500 kHz, and 1 MHz.

## Results

### Sensitivity field of the two-electrode configuration

**Fig. 4:**
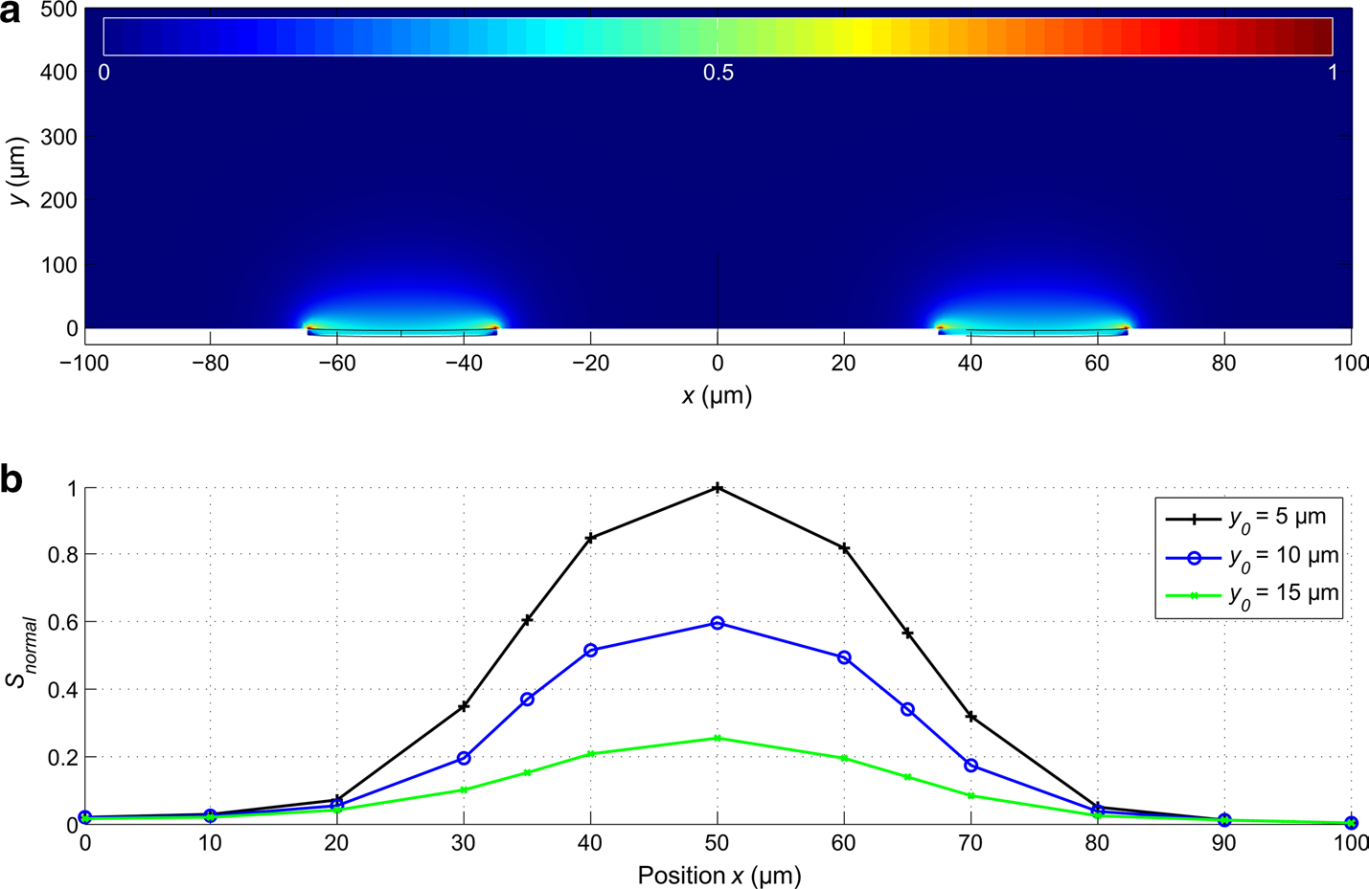
**a** Sensitivity field of the two-electrode system without a cell visualized in the *x*–*y* plane through the center of the electrodes, and **b** the normalized sensitivity along the* horizontal* position *x* at three different* vertical* positions *y* above the MEA base plate and electrodes. The normalized sensitivity (**b**) was calculated as the average over the volume of a cell at corresponding locations. Observe that here the* color scale* of sensitivity is between 0 and 1, as with a two electrode setup no negative values of *S* occur

In Fig. 4a is shown the simulated sensitivity field *S* of the two-electrode measurement configuration. In COMSOL Multiphysics, (2) was applied to compute the sensitivity field for each mesh element. Based on the lead field theory, we estimated the behavior of the impedance measurement system both qualitatively and quantitatively. As seen from Fig. 4a, measurement sensitivity of the two-electrode setup was positive in the entire analyzed volume, with the maxima at the edges of the microelectrodes where the current density was the highest due to the geometry effect of the edge. According to (1), a cell in a vicinity of a microelectrode caused a local decrease in the conductance, leading to an increase in the measured impedance. The absolute impedance increase depends on the location of the cell; the integral sensitivity in the volume occupied by the cell is crucial.

In Fig. 4b is shown the normalized integral sensitivity *S*_*normal*_ at different positions (*x*_0_ = 0, …, 100 µm, *y*_0_ = 5, 10 and 15 µm). The local sensitivity was the highest (normalized to 100%) in the region close to the center of the electrode at *x*_0_ = 50 µm, as expected. From Fig. 4b, it is seen that the highest sensitivity was reached with the cell located concentrically on the electrode. As the diameter of the cell was 20 µm, the cell extended above most of the electrode surface area, and the small region of maximum sensitivity near the edges of the electrode did not have much effect on the measurement. Moving away from the electrode, the sensitivity rapidly decreased as can be seen in Fig. 4b. At distances *x*_0_ >20 µm from the center of the electrode in the horizontal direction, sensitivity dropped below 40%, and down to 10% at *x*_0_ = 30 µm. It is to be noted that the simulation is free of scale, i.e., proportional changes are not affected by the size of the electrode.

The sensitivity field implies that adherent cells (*y*_0_ < 5 µm) influence the impedance measurement most, but the sensitivity was still at 60 and 25% of the maximum with a small gap of *y*_0_ = 10 and 15 µm, respectively, between the cell and the electrode. In theory, such sensitivity should be sufficient for the detection of non-adherent cells.

### Direct impedance simulations with the two-electrode configuration

**Fig. 5:**
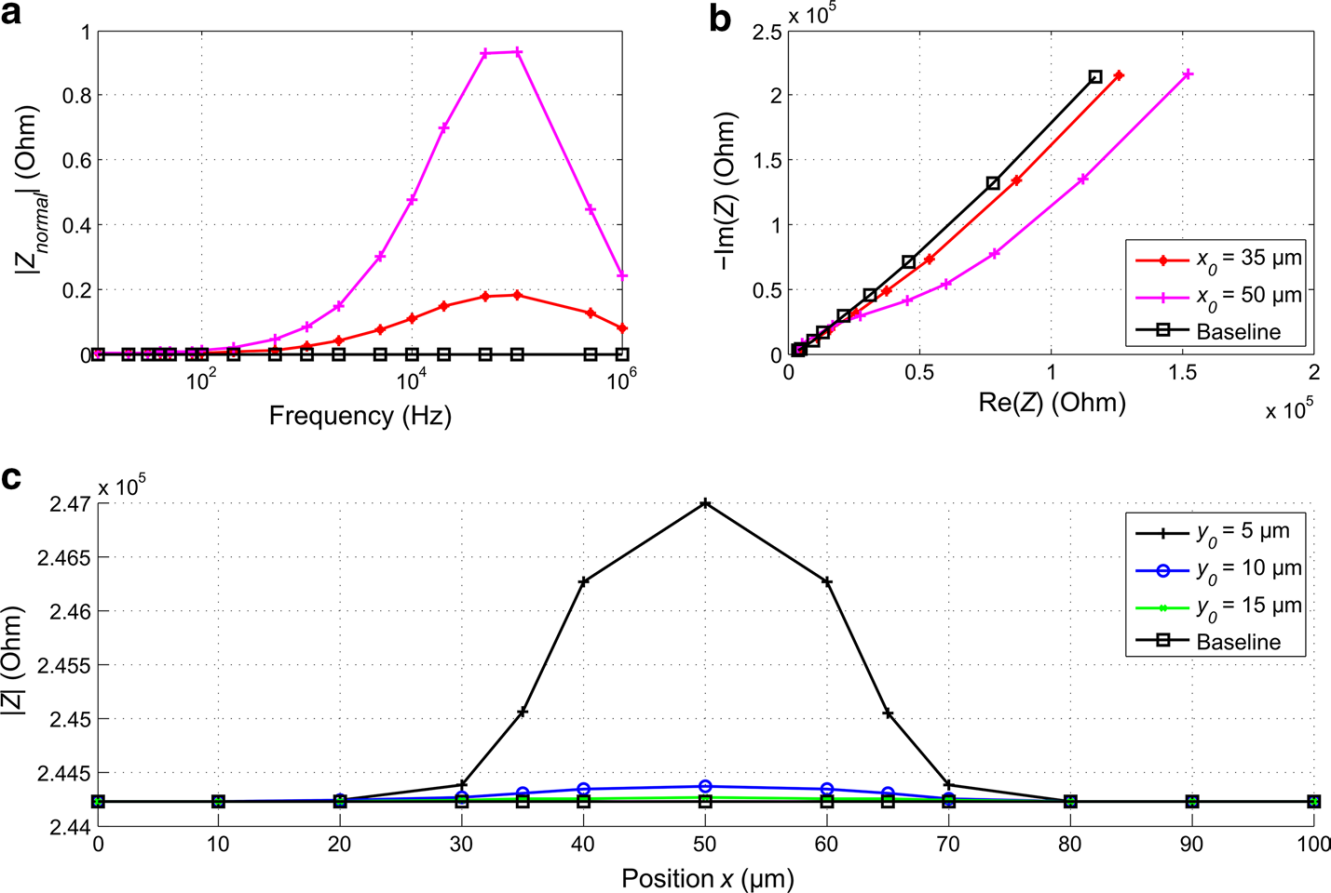
**a** The simulated normalized impedance magnitude spectrum and **b** locus for the two-electrode configuration. Baseline (**a**–**c**) is the impedance simulated without a cell. With the cell, positive sensitivity caused an increase in the impedance in the frequency range above 100 Hz in the normalized magnitude spectrum (**a**). The RC characteristic half ellipse is to some extent visible in the locus plot (**b**) at higher frequencies. **c** The impedance at 100 kHz shown as a function of the horizontal position of the cell for different vertical distances from the MEA base plate and electrodes. The microelectrode spanned between the points 35 and 65 µm, and the cell was 20 µm wide

To show the applicability of the lead field theory and sensitivity computations to MEA impedance measurement systems, direct simulations of impedance measurements were performed, and compared to the predictions based on the lead field analysis above. The results of the impedance simulations are shown in Fig. 5 with the spectrum and locus calculated from the simulated complex impedances for the two-electrode configuration. The normalized impedance is given by

7 $$|Z_{normal} | = \frac{{\left| Z \right| - |Z_{0} |}}{{|Z_{0} |}},$$

where *Z*_0_ is the impedance simulated without the cell.

As seen from Fig. 5a, the cell essentially affected the impedance spectrum at frequencies higher than 100 Hz with the maximum effect around 100 kHz. The impedance locus (Fig. 5b) shows a significantly higher real part caused by the cell at lower frequencies. The simulations confirmed the measurement results of Buitenweg et al. [3]. In Fig. 5b, the RC element of the cell membrane is manifested as the beginning of an ellipse at frequencies above 10 kHz.

To study the effects of the cell location on the impedance behavior at the frequency yielding the maximum deviation of the impedance from the baseline (i.e., from the impedance without the cell), in Fig. 5c is shown the impedance at 100 kHz simulated with the cell moving over the electrode along the horizontal axis. The impedance was the highest when the cell was concentrically above the electrode (Fig. 5c) since at that point the integral sensitivity over the cell volume was affected the most, as was predicted by the lead field analysis. The symmetry of the lead field *S* is observed as the symmetric behavior of the measured signal when the cell was moved. At *y*_0_ = 5 µm above the electrode surface and at a horizontal distance of *x*_0_ = 20 µm from the center of the electrode, the impedance dropped below 10% of the corresponding maximum. In comparison, the earlier predictions of the impedance (sensitivity of 40% at *x*_0_ = 20 µm) were overestimated.

Direct impedance simulations suggest that it may be difficult to detect non-adherent cells in the vicinity of the electrode with the two-electrode system. Whereas an adherent cell at *y*_0_ = 5 µm obviously influences the impedance, the impedance increase caused the appearance of a non-adherent cell at *y*_0_ ≥10 µm was less than 10%, and a cell not overlapping with the electrode did not notably affect the impedance. In this case, cell–electrode overlap would be necessary to affect the impedance spectrum sufficiently for reliable cell detection.

### Sensitivity field of the four-electrode configuration

**Fig. 6:**
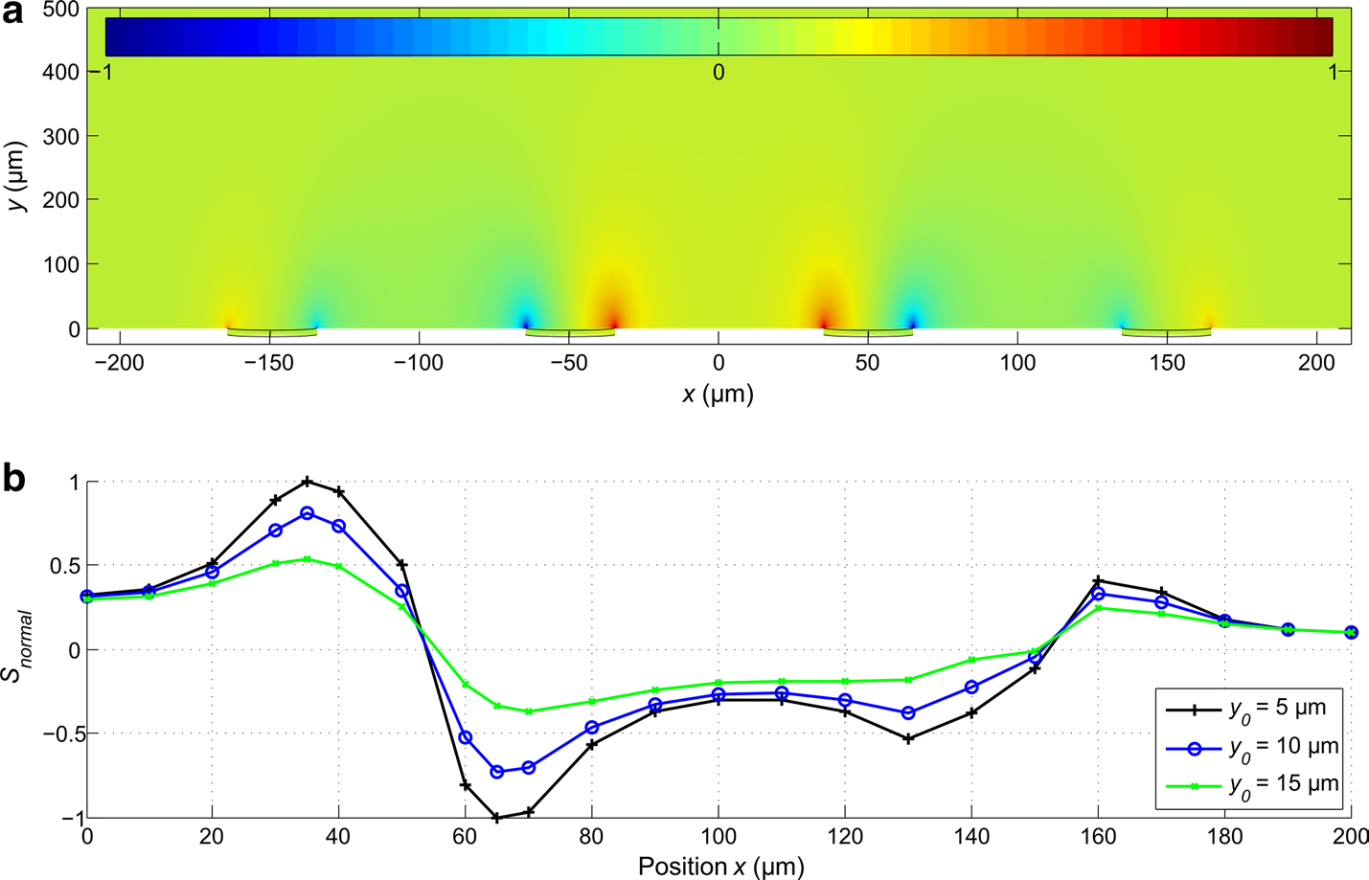
**a** The sensitivity field of the four-electrode system and **b** the normalized sensitivity at different positions on right half of the four-electrode setup (*x*_0_ ≥ 0 µm) (the *left side* would be a mirror image of **b**) as the average over the cell volume at corresponding locations. Observe that here the sensitivity color scale is between −1 and 1 as with a four electrode setup both positive and negative values of *S* can occur

In Fig. 6a is shown the sensitivity lead field *S* of the four-electrode configuration. In general, the sensitivity distribution is far more complex than in the two-electrode system (Fig. 5a). Now, three cases appear (c.f. Fig. 6a): *S* > 0 in the areas between the recording electrodes, and at the edges of the excitation electrodes most distant from the excitation electrodes; *S* < 0 in the areas between the recording and excitation electrodes; *S* = 0 at the center of the electrodes and at the boundaries between the areas of negative and positive sensitivity.

In Fig. 6b, the integral sensitivity in a partial volume of the cell is shown for several positions of the cell. The values are normalized with respect to the global peak and the volume. The highest sensitivity (normalized to 100%) was reached in the volumes at the edges of the recording electrodes at *x*_0_ = 35 and 65 µm. For the excitation electrodes, the sensitivity also shows peaks in the areas above the electrode edges, but with the absolute magnitudes at around 50%. Thus, with the observed volume on the center of the electrode, the affected integral sensitivity with the cell directly above an excitation electrode was small, since the affected volumes with positive and negative sensitivities are almost equal. Figure 6b shows that the sensitivity was around 50% at the centers of the recording electrodes at *x*_0_ = 50 µm, and almost zero at the centers of the excitation electrodes at *x*_0_ = 150 µm. In the areas between the electrodes at *x*_0_ = 0 and 100 μm, the sensitivity dropped to 30%.

Further, the sensitivity analysis showed that an adherent cell (*y*_0_ = 5 µm) influenced the measured impedance the most. With the four-electrode measurement setup, it should be possible to detect also non-adherent cells (*y*_0_ = 15 µm), since the sensitivity was still approximately 50%, compared to the sensitivity curve at *y*_0_ = 5 μm. This indicates that with the four-electrode system, also cells in the vicinity of MEA microelectrodes could be detected with reasonable certainty, unlike with the two-electrode system.

### Direct impedance simulations with the four-electrode configuration

**Fig. 7:**
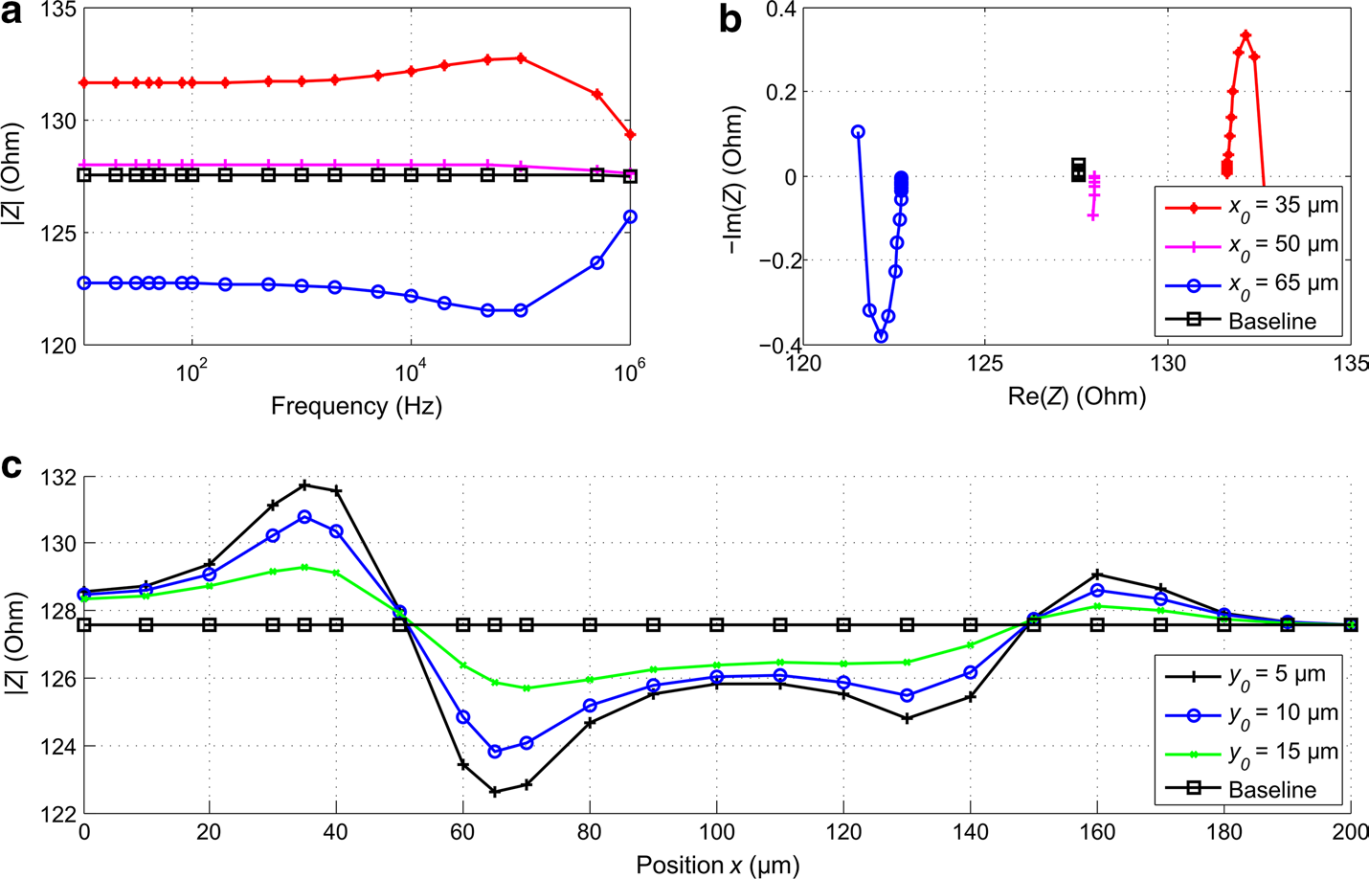
**a** The simulated normalized transfer impedance magnitude spectrum and **b** locus for the four-electrode configuration. Without the cell (baseline) the magnitude spectrum (**a**) is a *straight line.* The impedance locus (**b**) exhibits the characteristic *half ellipse*. **c** The impedance at 100 kHz shown as a function of the horizontal position of the cell for different vertical distances from the MEA base plate

The simulated transfer impedance magnitude spectrum and locus are shown in Fig. 7a, b for the four-electrode configuration, respectively. The baseline impedance, simulated without the cell, is seen to have been constant (Fig. 7a), as it should, since in an ideal four-electrode system the electrode impedance does not affect the measured signal. The spectrum acquired with a cell on the center of the electrode was almost equal to the baseline, and confirmed the prediction made by applying the lead field theory in “Sensitivity field of the four-electrode configuration” section. The cell located in the areas of positive or negative sensitivity caused an increase or decrease of the impedance, respectively. In this case, the cell equivalent RC element resulted in a typical waveform in the spectrum, where the positive or negative sensitivity resulted in a continuous increase or decrease in impedance, respectively, as also predicted by the lead field theory. The typical Cole half ellipse is visible in the locus plot in Fig. 7b. The orientation of the impedance locus is seen to be characteristic for the position of the cell. The negative and positive imaginary parts were characteristic for a cell located in a volume with negative or positive sensitivity, respectively. This indicates that the first estimate of the position of the cell can be made without the knowledge of the baseline impedance. In Fig. 7c is shown the impedance at 100 Hz simulated with the cell at different positions along the horizontal axis; this illustrates the influence of the cell located in the areas of different sensitivity. In Fig. 7c, the recording microelectrode spans between the points 35 and 65 µm, and the excitation microelectrode between 135 and 165 µm. As also predicted by the lead field theory, the cell influenced the impedance measurement the most when it was located at the edges of the microelectrodes at *x*_0_ = 35 or 65 µm. Similar behavior can be seen at the edges of the excitation electrodes, where the absolute impedance deviation was much weaker, approximately 40% of the maximum. Between the electrodes, the impedance was 20% at *x*_0_ = 0 µm, and 40% at *x*_0_ = 100 µm, which are in accordance with the lead field theory based predictions.

An adhered cell (*y*_0_ = 5 µm) affected the impedance the most. It can be seen in Fig. 7c that a non-adhered cell centered horizontally at *y*_0_ = 15 µm with a cell–electrode gap of 5 µm still caused an impedance deviation of approximately 37%, which can be easily detected. The simulated impedance values were slightly smaller than the sensitivity field would have implied, but the trends of the curves in Figs. 6b and 7c are very similar, thus confirming results from the lead field theory approach.

## Discussion

In this paper, we presented a simulation study of the application of the lead field concept as a tool for the optimization of MEA impedance measurements, including the electrode setup, analysis of cell detection, and visualization of the characteristics of electrode configurations. Despite its long history and popularity in the analysis of electrophysiological [19, 29–32] and thorax bioimpedance signals [23, 33, 34], the lead field concept has not been previously employed in the design and analysis of microelectrode systems for cell applications. Here, FEM simulations were used to compute the sensitivity fields of the two- and four-electrode measurement configurations to assess the impedance behavior of the systems. Direct impedance simulations in the presence and absence of a cell were performed to demonstrate the applicability of the lead field approach, and to illustrate the IS measurements with two- and four-electrode systems utilizing MEAs. The sensitivity fields of the two- and four-electrode setups differed, and the differences in the sensitivity fields of the two setups were as expected based on the earlier lead field simulations of human thorax and impedance tomography: The two-electrode setup exhibits only positive sensitivity values, whereas the four-electrode setup can exhibit both positive and negative values and more complex lead fields [23, 33, 34]. The different sensitivity fields of the two systems had clear implications on the possible effective designs of cell detection systems: The areas of highest sensitivities, as well as of zero sensitivity, are located profoundly differently with respect to the electrode locations in the two systems. For two-electrode setup, the lead field practically vanishes just above the electrode surface as the measurement sensitivity is very focused on the electrode surface. Analogously to the method presented in this paper, lead field theory can be employed in the design of more sophisticated MEA systems for cell applications.

Lead fields can be used to estimate the sensitivity distribution of the measurement configuration, and to compare and optimize measurement setups [34]. To estimate the influence of small conductivity changes, the sensitivity field is considered constant, for example, in the area next to the cell membrane, whereas for impedance simulations with different electrode configurations, the location of the cell can be assumed to remain constant for each cell–electrode constellation. The results from various electrode configurations can be compared in the sense of differences in the sensitivity fields, given that the impedivity of the medium is spatially and temporally constant. The measurement setup with the highest sensitivity for the specific measurement target provides the best results for the assessment of biological cells.

Because of the relatively high impedance of the microelectrodes, the effect of the RC element, modeling the cell membrane, on the impedance spectrum and locus was very small. If the impedance spectrum of the specific electrode–electrolyte interface was known, normalized magnitude spectrum would provide evidence of the presence or absence of a cell; even small impedance changes would be observable, if the cell was located in the areas with sensitivity slightly greater than zero.

Our simulations show that the choice of the proper electrode setup is essential, and that the lead field simulation is a potential tool for the assessment of the sensitivity and overall applicability of a particular electrode system. Our results indicate that the two-electrode configuration is suitable only for the detection of cells that are adhered onto the microelectrode: the two-electrode configuration can be used to detect cells that have by far settled down, or grow on the surface of the MEA electrodes. On the other hand, the four-electrode configuration enables the detection and observation of cells also in the vicinity of microelectrodes. The sensitivity distribution of a four-electrode measurement system is complex: the sensitivity is approximately zero at the center of the electrode, whereas at the opposite edges of a voltage sensing electrode, the sensitivity has opposite signs. Between the electrodes, the sign of sensitivity varies. Thus, a four-electrode configuration is suitable at least for systems in which the cells do not settle down on the surface of the MEA, for example, the cells may be flowing over the MEA, or if detecting the location of a cell between the electrodes is desired. With these considerations, both two- and four-electrode systems, with their own distinct merits, are suitable for cell detection. With both systems, the normalized impedance or the shape of the impedance locus can be used to detect cells.

The impedance estimations made here based on the sensitivity fields, did not always quantitatively fully meet the results of the direct impedance simulations. One reason for this is that we calculated and analyzed volume averages of the lead fields at various possible cell positions without the cell actually in the field, implying that the cell was modeled as homogeneous medium with spatially independent conductivity. Direct impedance simulations were done with a complex cell model to mimic biological conditions. The electrical characteristics of the cell membrane and the medium were included in the simulation to create an electrically inhomogeneous object with spatially distributed impedance. The cell changed its shape depending on its position, causing also the spatial distribution of the impedance to change. The lead field based analysis is valid as long as the object being measured does not too drastically change the current fields. To compute the impedance based on lead fields exactly, the exact geometrical impedance parameters would have to be considered. However, the analysis of the sensitivity field of a specific measurement setup helps to visualize the characteristics of the system in general. Moreover, as exact spatial impedance distributions of cells are not known in experimental setups, and depend on the conditions and properties of the cells, lead field analysis provides adequate estimates. Our simulations show that it is possible to assess the behavior of a measurement setup using the lead field approach even without detailed knowledge of the impedance characteristics of the cells. For the simulations, the parameters of the MEA electrodes can be determined with the help of equivalent circuit diagrams and curve fitting tools as described in this paper. Using the proposed approach, a MEA impedance measurement system can be designed, analyzed, and optimized.

The systems simulated in this paper are necessarily ideal, including the spatiotemporal properties of the medium and electrodes. In addition, many of the actual biological cells of interest are not spheres or spherical caps, as assumed here. Nevertheless, clearly defined and simplified simulations are necessary to gain the basic understanding of MEA IS measurement systems for cell applications. The fundamental qualitative phenomena observed in the simulation results will occur also in the real world MEA IS systems, although the quantitative output of a real world measurement system will differ from those observed in our idealistic simulations. We believe that the concepts and simulation results presented in this paper will give a real world cell detection and assessment system designers a firm basis for their work.

## Conclusions

We demonstrated the usefulness and power of the lead field concept and reciprocity in analyzing MEA impedance measurement systems for cell detection and assessment applications. We compared two- and four-electrode systems, illustrated their fundamentally different sensitivity fields, and commented on their proper and potential applications. The knowledge of the measurement sensitivity distributions allowed us to compare the efficiencies of different measurement setups, and would thus facilitate measurement setup optimization. The lead field concept is seen to enable targeted design of sensitivity fields for measurements tasks at hand for cell detection and assessment.

## Abbreviations

CPE: constant phase element
FEM: finite element method
IS: impedance spectroscopy
MEA: microelectrode array
MCS: Multi Channel Systems MCS GmbH
PBS: phosphate buffered saline
RC element: a simulation element consisting of a resistor (R) and capacitor (C)
